# Soluble transferrin receptor and immature reticulocytes are not useful for distinguishing iron-deficiency anemia from heterozygous beta-thalassemia

**DOI:** 10.1590/S1516-31802003000200012

**Published:** 2003-03-05

**Authors:** Gisélia Aparecida Freire Maia de Lima, Helena Zerlotti Wolf Grotto

**Keywords:** Soluble, Transferrin, Receptor, Iron, Deficiency, Anemia, Heterozygous, Beta-thalassemia, Reticulocytes, Receptor, Solúvel, Transferrina, Anemia, Ferropriva, Beta-talassemia, Heterozigótica, Reticulócitos

## Abstract

Iron deficiency and heterozygous beta-thalassemia are important causes of hypochromic-microcytic anemia. Two laboratory parameters are suggested for the differentiation of such anemia. High-fluorescence reticulocyte counts and soluble transferrin receptor levels were determined in iron-deficiency anemia patients (n = 49) and heterozygous beta-thalassemia patients (n = 43). There was no significant difference in high-fluorescence reticulocyte and soluble transferrin receptor values between the two groups, but a correlation was observed between high-fluorescence reticulocytes and soluble transfer-rin receptors in iron-deficiency anemia, probably due to increased receptor synthesis as a response to decreased iron content in erythrocytes.

## RESUMO

A deficiência de ferro e a beta-talassemia heterozigótica são importantes causas de microcitose e hipocromia. São propostos dois parâmetros laboratoriais para auxiliar na diferenciação entre essas anemias. O número de reticulócitos altamente imaturos e níveis do receptor solúvel da transferrina foram determinados em pacientes com anemia ferropriva (n = 49) e com beta-talassemia heterozigótica (n = 43). Não houve diferença significativa entre os valores de reticulócitos altamente imaturos e níveis de receptor solúvel da transferrina nos dois grupos, mas foi observada uma correlação entre reticulócitos altamente imaturos e níveis de receptor solúvel da transferrina no grupo com anemia ferropriva, provavelmente devido à um estímulo para a síntese do receptor em resposta à deprivação de ferro nos eritrócitos.

## INTRODUCTION

The soluble transferrin receptor is a truncated form of tissue receptor that circulates in serum as a complex of transferrin and its receptor.^1^ Studies indicate that the concentration of soluble transferrin receptor rises with enhanced erythropoiesis and iron deficiency.^2^

Immature reticulocytes are released from bone marrow in the event of enhanced erythropoietic activity. Transferrin receptors are present in reticulocyte membranes, but not in mature red cells. The younger the reticulocyte is, the greater the number of receptors per cell.^1^ Thus, a correlation between the number of immature reticulocytes and the soluble transferrin receptor concentration is expected.

## PATIENTS AND METHODS

We studied 43 patients with heterozygous beta-thalassemia and 49 patients with iron-deficiency anemia in order to determine the high-fluorescence reticulocyte count and soluble transferrin receptor concentration in the two groups, and whether these parameters could be used to distinguish heterozygous beta-thalassemia from iron-deficiency anemia. Fifty-seven non-anemic subjects were used as a control group.

Iron-deficiency anemia patients showed serum ferritin levels of under 30 ng/ml for men and 12 ng/ml for women (considered the minimum normal ferritin levels in our laboratory). Patients with hypochromic-microcytic anemia, hemoglobin A2 level over 3.4% and normal serum ferritin were considered to be heterozygous beta-thalassemia cases. The reticulocyte count and percentage of highly immature reticulocytes were obtained using a Cell-Dyn 3500 (Abbott — USA) analyzer. Reticulocytes were identified by a non-fluorescent method using new methylene blue as the dye. Fractions of reticulocyte immaturity were calculated on the basis of absorption intensity, and they were classified as mature, partially mature and highly immature reticulocyte fractions. Soluble transferrin receptor concentrations were estimated by an immunoenzymatic technique (Quantikine-R & D Systems — USA). The Kruskal-Wallis test was used for comparing the variables between groups. The correlation between high-fluorescence reticulocytes and soluble transferrin receptor was calculated via the Spearman correlation coefficient test. We considered p values equal to or lower than 0.05 to be significant. The capacity of the variables to differentiate between iron-deficiency anemia and heterozygous beta-thalassemia was studied by means of the receiver operating characteristic (ROC) curve.

## Results

The absolute reticulocyte count and soluble transferrin receptor levels were increased (p < 0.001) in heterozygous beta-thalassemia (mean ± standard deviation, SD: 160.1 ± 137.8 x 10^9^/liter and 27.1 ± 15.8 nmol/liter, respectively) and iron-deficiency anemia (mean ± SD: 93.7 ± 52.4 x 10^9^/liter and 50.2 ± 21.4 nmol/liter, respectively), in comparison with the control group (mean ± SD: 67.9 ± 17.6 x 10^9^/liter and 16.8 ± 3.1 nmol/liter, respectively). There was no significant difference in high-fluorescence reticulocyte values between the iron-deficiency anemia and heterozygous beta-thalassemia groups. The correlation between high-fluorescence reticulocytes and soluble transferrin receptor was weak and only reached significance (p = 0.025, r = 0.349) in the iron-deficiency anemia group. Soluble transferrin receptor presented better accuracy (87.6%) than high-fluorescence reticulocytes (54.1%) for distinguishing iron-deficiency anemia from heterozygous beta-thalassemia. High-fluorescence reticulocyte values of less than 8.3% showed 82% sensitivity and 30.2% specificity for recognizing iron-deficiency anemia patients. Soluble transferrin receptor values of more than 23 nmol/liter showed 100% sensitivity for recognizing iron-deficiency anemia patients, but only 69.4% specificity.

## DISCUSSION

The synthesis of surface transferrin receptor is proportional to the iron requirement of the cell, as a response to insufficient supply of transferrin iron.^3^ In addition, soluble transferrin receptor provides an assessment of erythropoiesis status, because the increase in soluble transferrin receptor concentration is proportional to erythroid marrow expansion.^1^ Our results confirm these concepts, as iron-deficiency anemia and heterozygous beta-thalassemia showed soluble transferrin receptor concentrations that were higher than in the control group. In iron-deficiency anemia patients, soluble transferrin receptor determinations were higher than in heterozygous beta-thalassemia, but an overlap between the two groups was observed. Gimferrer et al.,^4^ using the same discriminator value for serum ferritin as we did, observed high values for soluble transferrin receptor in heterozygous beta-thalassemia and heterozygous beta-thalassemia associated with iron-deficiency anemia patients. The authors concluded that soluble transferrin receptor was not useful in diagnosing the association of iron-deficiency anemia with heterozygous beta-thalassemia.

High-fluorescence reticulocytes have been described as a good discriminator between heterozygous beta-thalassemia and iron-deficiency anemia.^5^ Such difference was not observed in our study. The percentage of high-fluorescence reticulocytes was very similar in controls (mean ± SD: 6.2 ± 2.9%), heterozygous beta-thalassemia (mean ± SD: 7.3 ± 3.7%) and iron-deficiency anemia patients (mean ± SD: 6.9 ± 3.5%).

The correlation between high-fluorescence reticulocytes and soluble transferrin receptor in iron-deficiency anemia may be explained by a high concentration of transferrin receptor synthesized as response to decreased iron content in red blood cell progenitors.

## CONCLUSION

We conclude that soluble transferrin receptor and high-fluorescence reticulocytes are not useful for distinguishing heterozygous beta-thalassemia from iron-deficiency anemia patients, although they provide interesting data concerning erythropoietic activity.

## References

[1] Beguin Y (1992). The soluble transferrin receptor: biological aspects and clinical usefulness as quantitative measure of erythropoiesis. Haematologica.

[2] Kohgo Y, Nishisato T, Kondo H (1986). Circulating transferrin receptor in human serum. Br J Haematol.

[3] Cook JD (1999). The measurement of serum transferrin receptor. Am J Med Sci.

[4] Gimferrer E, Ubeda J, Remacha AF (1997). Serum transferrin receptor levels are "physiologically" high in heterozygous beta-thalassemia. Haematologica.

[5] Yoldi F, de Blas JM, Alvarez D, Alonso D, Rivera F, Rodriguez JM (1991). Automatización del recuento de reticulocitos. Sangre.

